# What’s New in Research during the Current Epidemic Wave of Eating Disorders?

**DOI:** 10.3390/jcm12123994

**Published:** 2023-06-12

**Authors:** Matteo Panero, Giovanni Abbate-Daga

**Affiliations:** Eating Disorders Center for Treatment and Research, Department of Neuroscience, University of Turin, Via Cherasco 11, 10126 Turin, Italy; matteo.panero@unito.it

The COVID-19 pandemic has caused physical health concerns and has significantly impacted mental health. Fear and anxiety have become widespread, as individuals grapple with uncertainty, isolation, and heightened stress levels due to factors such as the fear of contracting the virus, the uncertainty surrounding its consequences, and the disruptive changes to daily life. Among those susceptible to the detrimental effects of fear and anxiety are individuals with Anorexia Nervosa (AN). In fact, emotional dysregulation is a core psychological characteristic of AN and fear is a primary emotion described as elevated in individuals with AN. Persons suffering from AN typically have a distorted perception of their body shape and size, viewing themselves as overweight even when they are severely underweight. This perception fuels their fear, and they may resort to extreme measures such as fasting, excessive exercise, self-induced vomiting, or the abuse of laxatives to control their weight. These problems have certainly been exacerbated by the pandemic, as exercise-related compensation systems have been altered, greatly alarming patients. In AN, fear also relates to food presentation, which activates the biological stress circuitry: food choice (a relevant problem during the pandemic, particularly highlighted by the media and social networks which have further spread the phobia of “fattening” foods) in AN is highly determined by fear of weight gain, which is associated with elevated dorsal striatal brain response [1].

Therefore, the COVID-19 pandemic has significantly impacted individuals suffering from eating disorders (EDs), particularly concerning the worsening of self-harm behaviors and food-related and body-related fears. The combination of fear, anxiety, social isolation, disrupted routines, and increased stress levels experienced during the pandemic may have had a particular effect on the mental health of young individuals with AN, with both a sharp increase in hospitalizations and a high risk of self-harming behaviors, as described in a French nationwide population study [2]. Furthermore, body-related symptoms (i.e., body shape concerns, body checking, body avoidance) were higher after the onset of the pandemic for inpatients with severe anorexia nervosa when compared to inpatients hospitalized in the pre-pandemic period [3]. So, the first and one of the relevant effects of the pandemic was an increase in the onset of acute cases requiring hospitalization and a worsening of the symptoms of body and food psychopathology.

Furthermore, social distancing measures and periods of confinement associated with limited external social interactions may have intensified the fear response to social contact and feelings of loneliness and isolation, worsening the impact of uncomfortable social feelings in the vulnerable population. In fact, AN individuals’ proneness to fear and negative emotions may also involve an increased propensity to experience feelings of shame [4], often associated with the avoidance of social situations and fears related to the interpersonal sphere, which were intensified by the pandemic. Such characteristics have also led to the association of anorexia nervosa with autism spectrum disorders (ASD). A recent study has stated that early detection of ASD elements in individuals with eating disorders can greatly contribute to more appropriate and effective clinical care [5]. Thus, understanding the specific ASD-related features, such as sensory sensitivities, repetitive behaviors, and social communication difficulties, can help clinicians to provide a better staging of the disorder and tailor treatment plans and interventions accordingly, particularly in light of the emotional and isolation problems exacerbated by the pandemic in the years 2020–2022, the consequences of which are still visible today. Subjects with EDs and autistic traits may have particularly suffered the interruption of routines and compensations built up over the years. Subsequently, the re-opening period has all the characteristics to have been an important personal social stress for these individuals.

From a treatment perspective, during 2020 and 2021, the partial or total closure of services specialized in the treatment of EDs produced not only an increase in the number of hospitalizations of subjects suffering from AN but also a worsening of their clinical conditions, with admissions at lower BMIs and with more serious medical problems (i.e., electrolyte abnormalities, bradycardia, total fasting). AN, especially in its severe manifestations, entails significant difficulties for clinicians, leading to emotional responses such as fears about achieving successful treatment outcomes or regarding the potentially life-threatening nature of this condition. Consequently, implementing staging strategies becomes crucial to effectively recognize and address patients with severe characteristics, such as being extremely underweight. Toppino and colleagues [6] have studied different Body Mass Index (BMI) specifiers for severity and even proposed a novel “very extreme” BMI specifier for patients with a BMI ≤ 13.5; however, they eventually found that BMI is not useful in the staging of severity for inpatients with severe AN. This supports the notion that BMI is a clinical factor that should not be evaluated independently but in association with other medical and psychopathological characteristics to promote better staging in severe AN. In this sense, the lower BMIs found during hospitalizations in the years of the pandemic, taken as isolated data from the clinical context, do not constitute an index of greater severity. A multifactorial evaluation is more useful, as discussed in a recent comprehensive review that highlights the presence of complications in subjects suffering from EDs and low BMI [7]. Various medical complications have been associated with numerous physiological changes, resulting in reduced energy expenditure. These complications encompass cardiac, bone, obstetric, and gynecological alterations, as well as endocrine, gastrointestinal, hematological, electrolyte imbalances, and skin changes. The detrimental effects of severe malnutrition further perpetuate the eating disorder by exacerbating both physical and psychological discomfort during periods of malnutrition and refeeding. Awareness of current and real medical complications that occur in patients with severe AN and an informed care approach can help both clinicians and families to recognize the complexity of the illness and may lessen feelings of fear and helplessness. An unexpected positive effect of the pandemic—that can be evaluated in the long term—could derive from the fact that patients and families have been receiving more media attention and that information on EDs seems to have spread in the general population. Always keeping in mind the need for an in-depth evaluation, the accurate diagnosis of both AN itself and its accompanying comorbidities assumes significant importance. A recent scientific investigation [8] has examined the concordance between diagnoses formulated by clinicians specialized in eating disorders and those obtained through the valuation with the Structured and Clinical Interview for DSM-5 (SCID-5). Notably, the study raises concerns regarding recognizing comorbidities and associated symptoms in the diagnostic process. Some comorbidities, especially anxiety disorders, and PTSD, may maintain AN psychopathology, so it could be useful to recognize the presence of comorbid disorders and to specifically target them in the treatment. This is all the more relevant since the pandemic itself increased fear and anxiety and may have generated PTSD-like symptoms in some subjects. Lastly, during the pandemic, newly ill subjects more often experienced depressive symptoms and suicidal thoughts in the early stages of treatment. These mixed clinical characteristics are still present today and appear to be a new form of ED and depression in the very young. This severe manifestation of AN may induce a fear response in clinicians and especially caregivers about the risk of suicide. In a study involving 172 individuals with ED [9], it was observed that dissociation and suicidality exhibited a direct association: elevated levels of dissociation have the potential to amplify the risk of suicide. Factors such as bodily-related disturbances, depression, and anxiety may play a role in the relationship between dissociation and suicidality. Moreover, depression and anxiety were found to moderate the mediating influence of body image parameters between heightened dissociation and increased risk of suicide. Again, also in this case, the pandemic has been the “perfect storm”, as levels of anxiety and depression have greatly increased during the pandemic, particularly in adolescents, who are the subjects with a higher risk of developing EDs.

Finally, the treatment of eating disorders aims to achieve symptomatic remission in individuals who are often very physically and mentally distressed and who have also suffered in recent years due to the dreadful conditions that occurred during the COVID-19 pandemic. During that period, isolation, fear, or psychiatric comorbidities have been serious factors in developing mental suffering and in worsening previous psychopathology. In the final paragraphs, there will be a presentation of some new advancements that offer opportunities for optimizing the treatments; nevertheless, it remains crucial to highlight that effective evidence-based treatments for AN are multidisciplinary and integrated. These treatments often necessitate specific settings, such as intensive outpatient programs or inpatient and partial hospitalizations. Mairhofer and colleagues [10] have investigated the efficacy of integrative inpatient treatment for adolescents with anorexia nervosa. Their findings revealed that at the time of discharge, 23.2% of patients achieved “Full remission”, 31.3% achieved “Partial remission”, and 45.5% showed “No remission”. These findings support the notion that such difficult-to-threat illnesses necessitate further improvement in treatment approaches. The search for new treatments is also essential in response to the increase in cases and the psychopathological changes we are describing after the pandemic, which will see us increasingly engaged in the coming years with a post-pandemic ED epidemic.

One approach that has shown promise in addressing the fear of gaining weight in AN is in vivo body exposure therapy [11]. Overall, the fear of gaining weight in anorexia nervosa is a significant barrier to recovery. This form of therapy involves gradually exposing individuals to their feared stimuli, in this case, their bodies and weight gain. The therapy aims to help individuals to confront their fears and challenge the distorted beliefs associated with weight gain. Through repeated exposure, individuals learn to tolerate the distress associated with these situations and develop more realistic perceptions of their bodies. Another innovative therapeutic option is Virtual Reality (VR). A systematic review has explored inner body perception in AN [12], and the authors proposed a Regenerative Virtual Therapy that integrates VR with different technology-based somatic modification techniques and clinical strategies to regenerate a faulty bodily experience by stimulating the multisensory brain mechanisms and promoting self-regenerative processes within the brain. In relation to the worsening of body-related symptoms described after the pandemic [3], both these treatment techniques should be more widely considered and implemented in specialist EDS services.

Moreover, regarding psychotherapeutic treatments, which remain crucial in treatment pathways, new studies may also evaluate biological outcome indices such as Peripheral Brain-Derived Neurotrophic Factor (BDNF) concentration levels. In fact, a recent study on psychotherapy in various psychiatric disorders observed that BDNF concentration was higher after psychotherapy in two studies of ED patients [13]. In light of the growing interest that biomarkers are receiving in psychiatry, as well as due to the hope that the analysis of large quantities of data through Artificial Intelligence can give us new indications for personalized and more effective treatments, this study on BDNF could be an indicator for future insights into network studies.

Finally, the impact of the COVID-19 pandemic on individuals with EDs has highlighted the role of negative emotions and especially fear in worsening the bodily symptoms and hampering treatment pathways. The field of research is wide, and prospects for therapeutic alternatives are developing, leading to promising alternatives or integrations to the well-established multidisciplinary care pathways that are nowadays offered in such difficult-to-treat disorders.
